# The Satellite DNAs Populating the Genome of *Trigona hyalinata* and the Sharing of a Highly Abundant satDNA in *Trigona* Genus

**DOI:** 10.3390/genes14020418

**Published:** 2023-02-06

**Authors:** Jaqueline A. Pereira, Diogo C. Cabral-de-Mello, Denilce M. Lopes

**Affiliations:** 1 Laboratório de Citogenética de Insetos, Departamento de Biologia Geral, Universidade Federal de Viçosa, P.H. Rolfs Avenue, Viçosa 36570-900, Minas Gerais, Brazil; 2 Departamento de Biologia Geral e Aplicada, Instituto de Biociêcias/IB, UNESP–Universidade Estadual Paulista, 24 A Avenue, Rio Claro 13506-900, São Paulo, Brazil; 3 Department of Experimental Biology, Genetics Area, University of Jaén, Paraje las Lagunillas s/n, 23071 Jaén, Spain

**Keywords:** *c*-heterochromatin, genome, RepeatExplorer, Meliponini, bees

## Abstract

Among Meliponini species, *c*-heterochromatin can occupy large portions of chromosomes. This characteristic could be useful for understanding evolutionary patterns of satellite DNAs (satDNAs), although few sequences have been characterized in these bees. In *Trigona*, phylogenetically represented by clades A and B, the *c*-heterochromatin is mostly located in one chromosome arm. Here we used different techniques, including restriction endonucleases and genome sequencing followed by chromosomal analysis, to identify satDNAs that may be contributing to the evolution of *c*-heterochromatin in *Trigona*. Our results revealed a highly abundant ThyaSat01-301 satDNA, corresponding to about 13.77% of the *Trigona hyalinata* genome. Another seven satDNAs were identified, one corresponding to 2.24%, and the other six corresponding to 0.545% of the genome. The satDNA ThyaSat01-301 was shown to be one of the main constituents of the *c*-heterochromatin of this species, as well as of other species belonging to clade B of *Trigona*. However, this satDNA was not observed on the chromosomes of species from clade A, demonstrating that the *c*-heterochromatin is evolving divergently between species of clade A and B, as a consequence of the evolution of repetitive DNA sequences. Finally, our data suggest the molecular diversification of the karyotypes, despite a conservated macrochromosomal structure on the genus.

## 1. Introduction

Eukaryotic genomes are rich in multiple classes of repetitive DNAs, including satellite DNA (satDNA) and Transposable Elements (TEs) [1]. The satDNAs can represent large portions of the genomes and are characterized as tandem repeats which have a repeat unit length frequently larger than 100 bp [1,2]. Over the years, the use of different techniques to study repetitive DNA, such as *C_0_t*-DNA renaturation kinetics, restriction enzymes, chromosomal analysis, and more recently sequencing technologies combined with computational approaches, has allowed us to understand the structure and evolution of satDNAs in genomes [3,4]. This class of repetitive DNA is found primarily on constitutive heterochromatin (*c*-heterochromatin) domains and are predominant on chromosomal regions such as telomeres and pericentromeres [3,5]. SatDNA sequences are highly dynamic, and some molecular mechanisms (unequal crossing over, transposition, rolling circle replication and reinsertion) contribute to variations in the number of copies, the nucleotide base pair composition, and ultimately their evolution [3,4]. Usually, groups of related species share families of satDNA from a common ancestor that can evolve independently within each species, according to the Library Hypothesis model [6].

In stingless bees (Meliponini), studies about the evolution of heterochromatin and repetitive DNA sequences are limited [7,8,9,10,11]. Since Kerr et al. [12] first described the karyotype of Meliponini species, the study of chromosomes was mainly limited to the use of techniques such as C-banding and DAPI/CMA_3_ fluorochromes staining. This approach gives only superficial information about *c*-heterochromatin [13,14,15] but revealed variable patterns of chromosomal organization of *c*-heterochromatin in Meliponini. In most species, the *c*-heterochromatin is located in one of the chromosomal arms, frequently occupying the large extension of the chromosomes, and especially in *Melipona* representatives, the *c*-heterochromatin occupies almost the entire length of the chromosomes or is restricted to the pericentromeric region [15,16,17,18,19]. Recently, some studies using *C_0_t*-DNA and restriction endonucleases to isolate repetitive DNA sequence showed that the composition of *c*-heterochromatin is divergent among some subgenera and genera of Meliponini [7,8,9,10]. Furthermore, the molecular composition of *c*-heterochromatin was presented in detail for two species, *Melipona quadrifasciata* and *Melipona scutellaris*, revealing that quantitative and qualitative changes in the repetitive DNA library contributed to the evolution of the highly divergent *c*-heterochromatin of the genus *Melipona* [11].

The *Trigona* (Meliponini) is composed of 32 species, and based on morphological and biological characteristics and geographical distribution, it is divided into nine groups of species [20]. According to the molecular phylogeny proposed by Rasmussem and Camargo [20], *Trigona* can be divided into two major clades, A and B, with the last shared common ancestor dating from approximately 19 million years, Mya [21]. The study of the karyotypes of *Trigona* species showed that the chromosome number of 2n = 34 is conserved, except for in *Trigona braueri* (as *Trigona fulviventris*) with 2n = 32 [17,22]. Similar to most species of Meliponini, a high amount of *c*-heterochromatin is observed in *Trigona*, occupying one of the chromosomal arms [15,17,18,19,22,23,24,25,26]. The *c*-heterochromatin in *Trigona* was characterized only by fluorochrome staining, revealing the enrichment of G + C base pairs in few [22,24] or multiple chromosomes [25,26].

Since most Meliponini species have a frequent occurrence of large portions of *c*-heterochromatin on chromosomes, putatively as a result of massive amplification, studies on this group could contribute to broadening the knowledge about repetitive DNAs and their role in genome evolution. Here, our aim was to investigate repetitive DNA sequences and their relationship with the evolution of *c*-heterochromatin in *Trigona* species in a phylogenetic context. For this, we used different approaches, including restriction endonucleases and low-coverage genome sequencing followed by computational and chromosomal analysis. Our data showed one highly abundant satDNA as the main repetitive DNA that constitutes the *c*-heterochromatin of some species of this genus belonging to clade B, but not to clade A. This revealed the conservation of heterochromatin in related species, but also some degree of molecular differentiation of the chromosomes in the genus, despite the conservation of the macrochromosomal structure.

## 2. Materials and Methods

### 2.1. Genomic DNA Isolation and Digestion by Restriction Enzymes

Total genomic DNA from adults of *T. hyalinata* was extracted according to Waldschmidt et al. [27]. In order to obtain repetitive DNAs, we digested *T. hyalinata* genomic DNA with the restriction enzymes HinfI, EcoRI, Eco571, VspI, and BcnI (Thermo Fisher Scientific). For the reactions, we used 2.5 μL of DNA (100 ng/μL), 0.5 μL of enzymes (10 U/μL), and 2.0 μL of Buffer (10×) incubated in a water bath at 37 °C for 4 h. The restriction products were separated on a 1.2% agarose gel, and the result of the DNA restriction that showed a band pattern typical for tandem repetitive sequences was isolated and purified from the gel using the NucleoSpin^®^ Gel and PCR kit (Neumann-Neander-Str. 6-8·52355 Düren, Germany) following the manufacturer’s instructions.

### 2.2. Cloning, Sequencing, and Sequence Analysis

The isolated and purified bands were modified using the A-tailing procedure and were inserted in a pGEM-T Easy Vector (Promega) and used for the transformation of competent *Escherichia coli* cells (DH5α). We obtained a total of eight positive clones via PCR screening using the primers M13 Reverse (--48) and T7 Promoter in which the plasmids were sequenced in both directions using the Sanger sequencing method, and this was carried out by the Myleus Facility (Belo Horizonte, Brazil). The nucleotide sequences obtained from the sequencing were analyzed using the software Geneious v4.8 [28]. To verify if the obtained sequences had similarities with other sequences previously described, we searched the Repbase v20.10 [29] and GenBank/NCBI DNA databases using the BLAST tool [30].

After characterizing the repetitive sequence isolated by enzymatic digestion, we designed primers manually: Forward 5′-AAGTGATCAGAGGAGAACGAT-3′ and Reverse 5′-ATATAGCAATGTGGCGGCCA-3′. PCR reactions were performed using a 10 × PCR Rxn Buffer, 0.2 mM of MgCl_2_, 0.16 mM of dNTPs, 2 mM of each primer, 1 U of Taq Platinum DNA Polymerase (Invitrogen), and 100 ng/µL of genomic DNA. The PCR conditions included an initial denaturation at 94 °C for 5 min and 30 cycles at 94 °C (30 s), 55 °C (30 s) and 72 °C (80 s), and a final extension at 72 °C for 5 min. The PCR products were checked on a 1% agarose gel. The monomeric bands were isolated and used for reamplification for further protocols.

### 2.3. Genome Sequencing and Satellite DNA Sequence Analysis

For the genome sequencing, the genomic DNA of the *T. hyalinata* was obtained using the DNeasy kit (Qiagen Inc., Valencia, CA, USA) according to the manufacturer’s protocol. The sequencing of the genome was performed using Illumina Hiseq 2000 (Inc., San Diego, CA, USA), and we obtained paired-end reads (2 × 150 bp) using the service of Novogene (Beijing, China). The genomic reads were deposited in GenBank under accession number SRX17748075. To identify the abundant satDNAs populating the genome of *T. hyalinata*, we used RepeatExplorer2 (Galaxy Version 2.3.7), available on the public platform https://repeatexplorer-elixir.cerit-sc.cz/galaxy (accessed on 4 August 2021 ). These analyses followed the protocol suggested by Novák et al. [31]. After grouping by RepeatExplorer2, the satDNAs were identified using the TAREAN tool [32]. All sequences were analyzed to identify possible similarities with satDNA previously identified by comparison with sequences deposited on Repbase v20.10 [29] and in the GenBank/NCBI DNA databases with the BASTn tool [30]. The abundance and divergence of each satDNA was estimated by means of RepeatMasker [33] using the Cross_match. The abundance of each satDNA was estimated based on its proportion in the genome, so the sum of the mapped nucleotides belonging to one specific satDNA was divided by the total number of nucleotides in the library. The divergence of the satDNAs was determined using the Kimura 2-parameter (K2P) using the calcDivergenceFromAlign.pl script in software RepeatMasker [33]. Finally, we identified a possible homology between satDNA families using the software Geneious v4.8 [28]. The sequences were classified as families and grouped in superfamilies according to Ruiz-Ruano et al. [34]. Finally, the sequences were named according to Ruiz-Ruano et al. [34], considering the abundance.

After the genomic analysis, we found another satDNA with considerable abundance in *T. hyalinata*. This satDNA was isolated using the primers: Forward 5′- CAGATGCGATATCTCGTCGAT-3′ and Reverse 5′-CCGAGCTTTCTCGCAGTTTC-3′, following the PCR reaction described above for the satDNA isolated by restriction enzyme digestion.

### 2.4. Fluorescence In Situ Hybridization (FISH)

Mitotic chromosomes were obtained according to Imai et al. [35] from brain ganglia of postdefecating larvae of different species of *Trigona* from clades A and B (Table 1, Figure S1). FISH followed the protocol described by Pinkel et al. [36] with modifications. First, the slides were treated with RNase A (100 µg/mL) and kept at 37 °C for 1 h. Then, a wash was performed in 2 × SSC for 5 min and the slides were incubated in a solution containing 5 µg/mL of pepsin in 0.01 N HCl for 10 min. After, the slides were washed in 2 × SSC for 5 min and dehydrated in alcoholic series at the following concentrations, 50%, 70% and 100%, for 2 min each. For the denaturation of the metaphase chromosomes, we used 70% formamide/2 × SSC for 5 min. The repetitive sequence isolated from the *T. hyalinata* genome was labeled using an indirect method through PCR with digoxigenin-11-dUTP (Roche, Mannheim, Germany). For probe hybridization in the chromosomes, the slides were kept overnight at 37 °C. The slides were washed in 2 × SSC, and for signal detection, we used anti-digoxigenin-rodamine (Roche Applied Science, Mannheim, Germany) for 1 h at 37 °C. Finally, the slides were washed in 4 × SSC/Tween and dehydrated in an Ethanol series of 50%, 70%, and 100%. The chromosomes were counterstained with 4′,6-Diamidino-2-Phenylindole, Dihydrochloride (Sigma-Aldrich, Darmstadt, Germany). The metaphases were captured in an Olympus BX53F microscope equipped with an Olympus MX10 camera using the CellSens imaging software.

## 3. Results

### 3.1. Extensive Amplification of a satDNA Superfamily on the Genome of T. hyalinata

**Figure 1:**
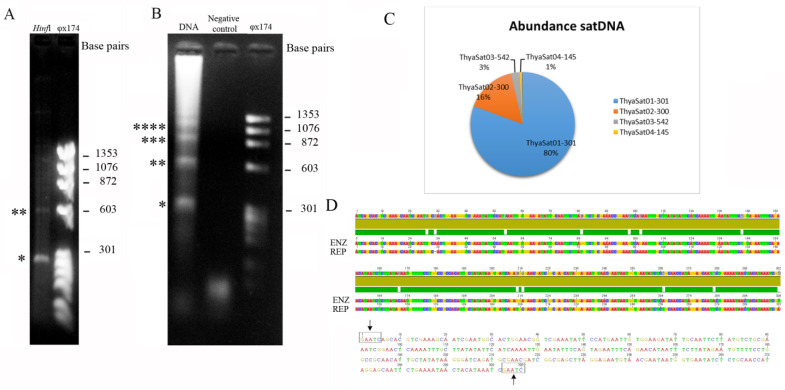
Satellite DNAs populating the genome of *T. hyalinata*. (**A**) Agarose gel showing the enzymatic digestion with HinfI that revealed occurrence of ladder pattern; one asterisk (monomer) and two asterisks (dimer). (**B**) Agarose gel after PCR amplification of ThyaSat01-301 showing the ladder pattern; quantity of asterisks corresponds to monomer multiples. (**C**) Relative abundance of the four most abundant satDNAs identified by RepeatExplorer. (**D**) Upper panel shows the alignment of consensus sequences obtained by restriction enzyme (ENZ) and by RepeatExplorer (REP) for ThyaSat01-301; note that the sequences are highly similar. The lower panel shows the consensus sequence from cloned fragments, and arrows indicate the restriction sites. * monomer, ** dimer, *** trimer and **** tetramer.

The enzymatic digestion of genomic DNA with HinfI allowed us to identify one repetitive DNA sequence from the genome of *T. hyalinata*; this was the only restriction enzyme that gave a ladder-like pattern, which represents one of the characteristics of a putative authentic tandem repeat (Figure 1A). Two fragments of approximately 300 bp and 600 bp were isolated. The sequence analysis from the cloned fragments revealed a sequence with 302 bp and a percentage of A + T = 63.2% with a repetitive pattern since the fragment with about 600 bp corresponds to two monomers. PCR assays for the sequence also revealed a ladder pattern, characteristic for in tandem repeats (Figure 1B). As we did not identify any correspondence of these fragments with other sequences of repetitive DNA previously characterized (TEs for example) and because they presented repetitively in tandem by the genome, we classified it as a satDNA.

Through RepeatExplorer clustering analysis, we identified eight satDNA families (deposited on GenBank under accession numbers OP559492-OP559499) which corresponded together to 16.565% of the *T. hyalinata* genome considering the characterized repetitive fraction (Table 2). These satDNAs monomer sequences ranged in length from 145 bp to 542 bp, and the A + T content of the sequences ranged from 55.9% to 66.1%. The homology analysis among the eight *T. hyalinata* satDNA families revealed an identity higher than 50% between some of them, so that it was possible to group some families into two superfamilies. The superfamily (SF1) was represented by ThyaSat01-301 and ThyaSat02-300, and the second superfamily (SF2) was represented by ThyaSa04-145, ThyaSat06-145, ThyaSat07-145, and ThyaSat08-145. In SF1, we noticed a difference of one base pair between the length of the monomer of consensus sequences for the two families, while in SF2, the monomers for the four satDNAs families had the same length. This indicates that the divergence between them was mainly due to nucleotide substitution, such as point mutation events (Table 2).

ThyaSat01-301 was the most abundant satDNA corresponding to 13.774% of the genome, which was about 83% of the characterized satDNAs (Table 2, Figure 1C). Furthermore, the ThyaSat01-301 corresponds to the satDNA isolated with the restriction enzyme HinfI (Figure 1D). Although much less abundant than ThyaSat01-301, another satDNA also showed relevant abundance in the *T. hyalinata* genome, the ThyaSat02-300, corresponding to 2.246% (14% of the satDNA content) of the genome. These two satDNA families were members of SF1 and together represented 97% of the satDNA content of the species genome (Table 2, Figure 1C). The other six satDNAs had a very low abundance, representing together only 0.545% of the genome. The average nucleotide K2P divergence of the eight satDNA families was 13.39%, ranging from 5.5% to 20.59% (Table 2).

**Figure 2:**
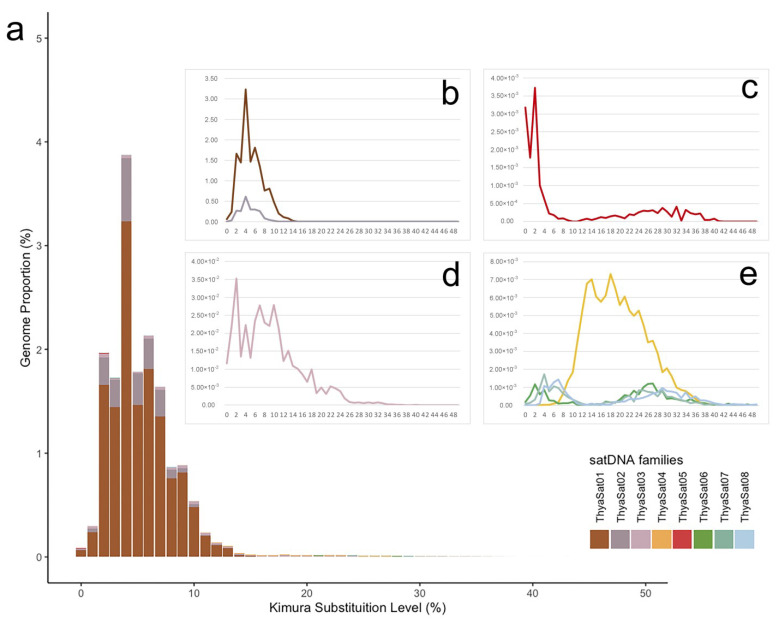
Landscape (abundance *versus* divergence) for the satDNAs identified on the genome of *T. hyalinata* through analysis of RepeatExplorer. The abundance and divergence were estimated by RepeatMasker. (**a**) General landscape for all satDNAs. (**b**–**e**) Show landscapes in more detail for specific satDNA families; (**b**) members of SF1, ThyaSat01-301, and ThyaSat02-300; (**c**) ThyaSat05-291; (**d**) ThyaSat03-542; (**e**) members of SF2, ThyaSat04-145, ThyaSat06-145, ThyaSat07-145, and ThyaSat08-145.

The analysis of landscapes (abundance versus divergence) evidenced a more recent amplification of the members of SF1 in comparison to other satDNAs, as it had a high abundance with lower divergence (K2P) (Figure 2a,b). A low K2P was also observed for ThyaSat05-291, but with no amplification pattern (Figure 2c). For the ThyaSat03-542 variable, K2P was noticed (Figure 2d). Finally, for the members of SF2, the distinct peaks of abundance in variable K2P for ThyaSat04-145 and the other members, i.e., ThyaSat06-145, ThyaSat07-145, and ThyaSat08-145 suggested that these families were under distinct patterns of homogenization/amplification (Figure 2e).

### 3.2. A Phylogenetically Conserved satDNA on Heterochromatin of Trigona Species

**Figure 3:**
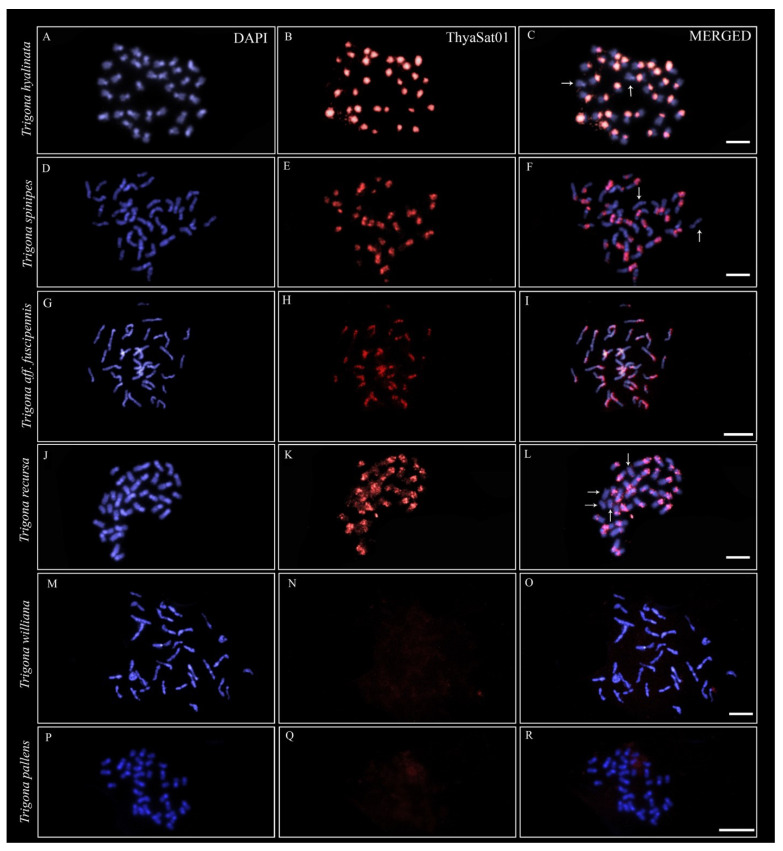
Chromosomal distribution of ThyaSat01-301 detected by FISH in *Trigona*. (**A**–**C**) *T. hyalinata*; (**D**–**F**) *Trigona spinipes*; (**G**–**I**) *Trigona* aff. *fuscipennis*; (**J**–**L**) *Trigona recursa;* (**M**–**O**) *Trigona williana*; (**P**–**R**) *Trigona pallens*. (**A**–**L**) Clade B representatives, (**M**–**R**) clade A representatives. Blue corresponds to chromosomes stained with DAPI and red corresponds to FISH signals. The arrows correspond to the chromosomes where no FISH signals were observed. Note differences in the intensity of hybridization signals between species, with occurrence of scattered signals for *Trigona fuscipennis* and *Trigona recursa*. Bar = 5 μm.

**Figure 4:**
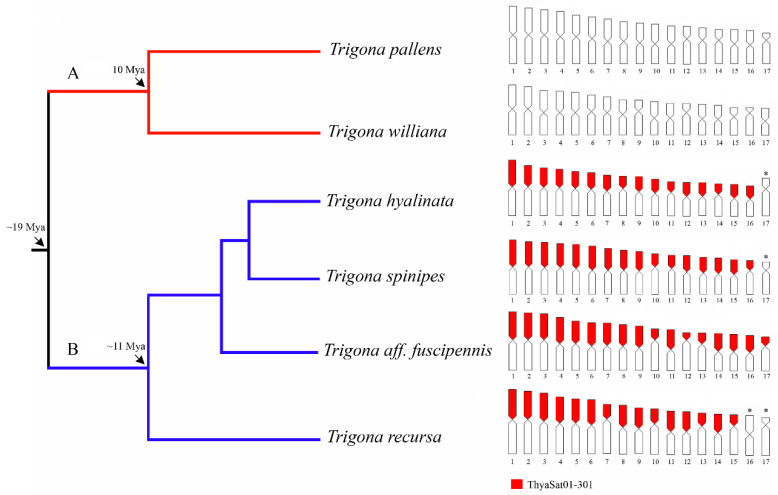
Simplified phylogeny for *Trigona* species based on Rasmussem and Camargo [20], Rasmussen and Cameron [21]. The ideograms on the right show the distribution of ThyaSat01-301 on the *Trigona* karyotypes. (*) Chromosomes in which no signs of ThyaSat01-301 were observed. Note that ThyaSat01-301 is located on the heterochromatic arm of chromosomes in clade B species (blue), but it is not present in clade A species (red).

We analyzed the chromosomal distribution of the main satDNA isolated from the *T. hyalinata* genome (ThyaSat01-301) in the other six species of *Trigona* belonging to clades A and B, and we observed positive FISH signals on four of them, all from clade B (Figure 3 and Figure 4). According to previously published data, the *c*-heterochromatin for these species is predominant in one of the chromosomal arms [17,26]. The *c*-heterochromatin is easily observed by DAPI staining as more brightness regions after formamide FISH treatment (Figure 3A,D,G,J,M,P). Thus, we observed that the ThyaSat01-301 satDNA was exclusively enriched on the *c*-heterochromatin region of the chromosomes (Figure 3).

In *T. hyalinata*, the signals formed large blocks occupying all the *c*-heterochromatin of almost all chromosomes, except the *c*-heterochromatin of one chromosome pair (Figure 3C). Similarly, in *T. spinipes* (Figure 3F), *T. aff. fuscipennis* (Figure 3I), and *T. recursa* (Figure 3L), the signals also coincided primarily with *c*-heterochromatic regions. In *T. spinipes* (Figure 3F) and *T. recursa* (Figure 3L), two and four chromosomes lacked signals, respectively. All of these species with positive FISH signals belonged to clade B, and no signal was identified for *T. williana* (Figure 3N) and *T. pallens* (Figure 3Q), which belonged to clade A.

**Figure 5:**
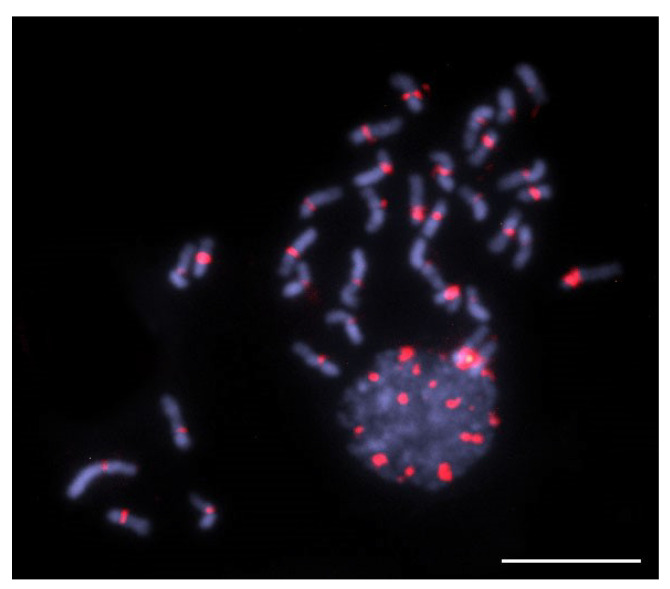
Chromosomal distribution of ThyaSat02-300 (red) detected by FISH in the chromosomes (blue) of *T. hyalinata*. Note that the signals for ThyaSat02-300 are much more restricted to the pericentromeric region in comparison to the signals observed for ThyaSat01-301 that occupy the whole extension of heterochromatin (Figure 3A–C).

The chromosomal mapping of the second most abundant satDNA (ThySat02-300) that belonged to SF1 on the *T. hyalinata* chromosomes revealed signals on the pericentromeric regions of most chromosomes, but it was not extended to the whole heterochromatin of chromosome arms like ThyaSat01-301 was (Figure 5).

## 4. Discussion

One of the most striking characteristics of bees from the Meliponini tribe is the high abundance of *c*-heterochromatin [16,17,19], as noticed on the *Trigona* species selected for this study. Most species share the same chromosome number and heterochromatin distribution on one arm of most chromosomes, and this is the commonly observed pattern. Recently, methods for repetitive DNA studies, as were used here, have allowed researchers to reveal differences in the karyotypes of Meliponini representatives, mainly on the *c*-heterochromatin. Even among congeneric species such as *Trigona*, relevant differences have been noticed, suggesting that the repetitive DNA in this group has undergone intense differentiation processes and can be an important tool for evolutionary genomic and karyotypic studies [11].

Here, we were able to identify eight satDNAs in the genome of *T. hyalinata*. The most abundant, ThyaSat01-301, was A + T enriched, similar to that observed on most satDNAs characterized on insects so far [37]. According to the RepeatExplorer analysis, ThyaSat01-301 satDNA was the most abundant (13.774% of abundance) on the *T. hyalinata* genome, and it was possible to be identified by enzymatic digestion forming a ladder-like pattern, reinforcing its true tandem organization. The ThyaSat01-301 formed large blocks that virtually covered the heterochromatin of most chromosomes of the species and was shared with some other *Trigona* representatives from clade B, suggesting that this is the main component of the *c*-heterochromatin in these species. This idea was supported by the high abundance of ThyaSat01-301 on the genome of *T. hyalinata*, corresponding to 13.774% of its genome.

The prevalence of one specific repetitive DNA occupying a large part of *c*-heterochromatin also seems to be recurrent for some genera of Meliponini, as already reported for *Tetragonisca* [9] and *Melipona* [10,11]. In some insects, the presence of one or more highly abundant families in the genome can also be observed in *Triatoma infestans* [38], *Hippodamia variegata* [39], *Holhymenia histrio* [40], and *Tribolium castaneum* [41]. However, these studies are based on the analysis of isolated species, so it is not possible to propose whether this characteristic also extends to other species belonging to the same subgenus or genus. Furthermore, for some insects, the most abundant satDNA may represent variable amounts of the genome [34,42,43,44]. In the case of *Trigona* and the previously studied *Melipona* [7,10], the high amount of heterochromatin suggests that the massive amplification of a certain satDNA seems to be a trend in Meliponini and occurs independently in each genus, subgenus, or specific clades. For ThyaSat01-301 and the other member of SF1, i.e., ThyaSat02-300, the high abundance and low divergence suggest that this amplification was more recent in comparison to other satDNAs families in the *T. hyalinata* genome. Although both families were amplified on the genome of *T. hyalinata*, the ThyaSat02-300 had experienced much less amplification, as evidenced by genome abundance and chromosomal location, being restricted to the pericentromeric region. The FISH data corroborated clearly that these two satDNAs were true different satDNAs, although some degree of similarity was shared. Considering that the occurrence of heterochromatin on pericentromeres as well as the satDNAs is more common and ancestral, we speculate that the satDNA ThyaSat02-300 was ancient and the ThyaSat01-301 was a derived satDNA able to spread on chromosome arms’ heterochromatin.

According to the phylogeny proposed by Rasmussen et al. [20], *Trigona* species are divided into two clades, A and B. Our FISH results revealed ThySat01-301 signals exclusively on chromosomes of representatives of the clade B species *T. hyalinata*, *T. spinipes*, *T. recursa*, and *T. aff. fuscipennis*. The sharing of the same satDNA between these species suggests an ancient origin that was possibly present in the common ancestor of this clade, approximately 11 Mya [21]. Although, we cannot completely rule out that this satDNA was also present in species of clade A (*T. williana* e *T. pallens*) in a very low proportion that was not detected by the FISH analysis. In any case, our data clearly evidence that the *c*-heterochromatin is evolving in a divergent manner between species of clade A and B, achieving different molecular compositions. This suggests that the amplification of heterochromatin in the genus, which had a similar distribution among species of clade A and B, occupying one entire arm of most chromosomes, occurred independently, or it was completely differentiated by mutational processes after ancestral amplification. This is a similar case to observed in the genus *Melipona*, in which the analysis of repetitive DNA revealed that the composition of *c*-heterochromatin was divergent between subgenera, suggesting more than one round of amplification of *c*-heterochromatin [7,8,10].

Among insects, analyses using high-throughput sequencing data and molecular cytogenetics clearly illustrate the evolutionary dynamics of satDNA over time [45,46,47]. Most satDNAs are highly dynamic and follow the principles of the Library Hypothesis, which predicts that sister species share a set of satDNA from the common ancestor and certain members of the library may be differentially amplified, appearing as the main component of satDNAs, while others occur at a low abundance, leading to distinct profiles of satDNA between species [6,45]. In *Trigona*, the difference in *c*-heterochromatin composition between species of clade A and B, evidenced by the presence of FISH signals for ThySat01-301 satDNA on representatives of clade B and absence on clade A, illustrate very well the predictions of the evolution of the satDNA library that differentiated approximately 19 Mya [21].

In *Trigona*, both intraspecific and interspecific variations were demonstrated in the number and location of 18 rDNA sites [25,26,48]. Interspecific variations occurred mainly between species of clade A and B. Thus, in clade A, the ribosomal sites were located in the interstitial region of the chromosomes, and in clade B, they were mainly placed on the terminal region of the chromosomes [48]. According to these previous works, the genomic dispersion of rDNA sites can be facilitated by the interaction with repetitive sequences located in *c*-heterochromatin. Furthermore, the molecular mechanisms act on the spreading of satDNA monomers, as the transposition and replication of extrachromosomal circles may be related to the dispersion of rDNA sites [3,26]. Although less variable, the chromosomal distribution of other repetitive DNAs are also variable in *Trigona* species, such as the microsatellite (GA)_15_ [25,26]. Thus, these cytogenetic data along with the *c*-heterochromatin divergence between clade A and B observed here highlight the genomic differentiation on *Trigona* species, despite the conservation of the diploid number (2n = 34).

In summary, we revealed that satDNA occupy a large part of *c*-heterochromatin in species of *Trigona* belonging to the B clade, showing that the heterochromatin is predominantly constituted by a single satDNA. Furthermore, we demonstrated that *c*-heterochromatin may be evolving in a divergent way between species of clade A and B, as a consequence of the amplification of different repetitive DNA sequences after species divergence. These data, summed using the previous study of other few species of Meliponini [11], reinforce the amplification and high dynamism of *c*-heterochromatin in this group of bees, highlighting the relevance to use these insects for other studies focused on *c*-heterochromatin evolution.

## Figures and Tables

**Table 1:** *Trigona* species and respective collection sites.

Species	Species Group *	Clades *	Sample Locality
*Trigona williana*	*“fulviventris”*	A	Altamira, Pará
*Trigona pallens*	*“pallens”*	A	Altamira, Pará
*T.hyalinata*	*“spinipes”*	B	Viçosa, Minas Gerais
*Trigona spinipes*	*“spinipes”*	B	Ribeirão Preto, São Paulo
*Trigona* aff. *fuscipennis*	*“fuscipennis”*	B	Florestal, Minas Gerais
*Trigona recursa*	*“recursa”*	B	Januária, Minas Gerais

* According to Rasmussem and Camaron (2008).

**Table 2:** Main attributes of satDNAs characterized from *T. hyalinata*.

Satellite DNA	SF	ML	A + T%	Divergence	Abundance
ThyaSat01-301	1	301	63.5	5.74%	13.774%
ThyaSat02-300	1	300	62.7	5.50%	2.246%
ThyaSat03-542		542	66.1	8.99%	0.370%
ThyaSat04-145	2	145	55.9	20.05%	0.110%
ThyaSat05-291		291	58.8	9.26%	0.016%
ThyaSat06-145	2	145	61.4	20.13%	0.015%
ThyaSat07-145	2	145	58.6	16.87%	0.017%
ThyaSat08-145	2	145	58.6	20.59%	0.017%
Total					16.565%

SF = superfamilies, ML = monomer length, A + T% = A + T sequence content.

## Data Availability

Not applicable.

## Supplementary Materials

The following supporting information can be downloaded at https://www.mdpi.com/article/10.3390/genes14020418/s1: Figure S1: Map of Brazil showing the collection sites for the *Trigona* species used in this study.
