# Supplementary figures and images for: The Satellite DNAs Populating the Genome of Trigona hyalinata and the Sharing of a Highly Abundant satDNA in Trigona Genus
Source: Genes (Basel). 2023 Feb 6;14(2):418. doi: 10.3390/genes14020418 (PMC9957317; doi:10.3390/genes14020418)

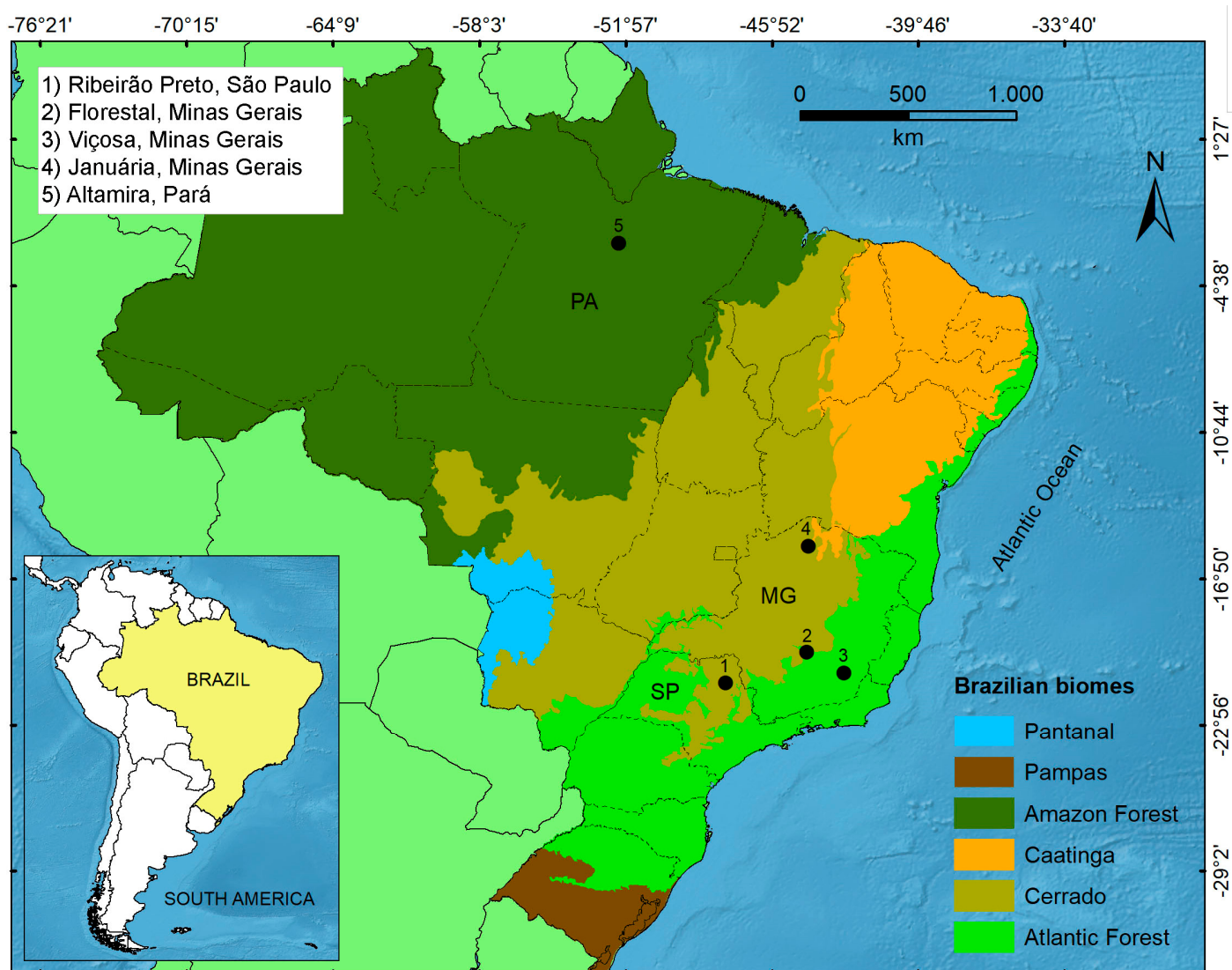

**Figure S1.** Map of Brazil showing the collection sites for the *Trigona* species used in this study.

Supplement: Supplementary file 1 [file genes-14-00418-s001.zip › genes-2043789-supplementary.pdf]
